# Habitat-Specific Patterns of Tick-Borne Pathogens in Urban and Suburban Landscapes

**DOI:** 10.3390/pathogens15040376

**Published:** 2026-04-01

**Authors:** Jana Radzijevskaja, Justina Snegiriovaitė, Asta Aleksandravičienė, Saulius Bernotas, Indrė Lipatova, Dalytė Mardosaitė-Busaitienė, Miglė Razgūnaitė, Algimantas Paulauskas

**Affiliations:** Faculty of Natural Sciences, Vytautas Magnus University, K. Donelaičio Str. 58, LT-44248 Kaunas, Lithuania; justina.snegiriovaite@vdu.lt (J.S.); asta.aleksandraviciene@vdu.lt (A.A.); saulius.bernotas@vdu.lt (S.B.); indre.lipatova@vdu.lt (I.L.); dalyte.mardosaite-busaitiene@vdu.lt (D.M.-B.); migle.razgunaite@vdu.lt (M.R.); algimantas.paulauskas@vdu.lt (A.P.)

**Keywords:** tick-borne pathogens, *Ixodes ricinus*, urban and suburban landscapes, Lithuania

## Abstract

Tick-borne diseases are an increasing public health concern in Europe, driven by climate change, landscape transformation, and expanding human activity. Urban green spaces provide suitable habitats for ticks and increase human exposure to tick-borne pathogens (TBPs), yet habitat-specific patterns in urban and suburban environments remain poorly characterized. This study examined tick distribution and TBPs prevalence across 11 urban and suburban sites in Kaunas County, Lithuania. A total of 1539 questing *Ixodes ricinus* ticks were collected and screened by real-time PCR for *Borrelia* spp., *Anaplasma phagocytophilum*, *Neoehrlichia mikurensis*, *Rickettsia* spp., and *Babesia* spp., with further species identification by sequencing. *Borrelia* spp. were most prevalent (24.43%), followed by *Rickettsia* spp. (7.60%), *N. mikurensis* (6.63%), *A. phagocytophilum* (3.64%), and *Babesia* spp. (2.53%). Tick density, pathogen prevalence, and species diversity varied among habitats, with higher values in forested and ecotonal areas, but notable infection rates were also observed in managed urban sites. Co-infections occurred in 18.8% of infected ticks. Our findings demonstrate that the circulation of TBPs in urban and suburban landscapes is shaped by local habitat features, host communities, and management intensity, highlighting the need for habitat-specific risk assessment in urban planning and public health.

## 1. Introduction

Tick-borne diseases represent a growing public health concern across Europe, driven by the combined effects of climate change, land-use modification, wildlife host dynamics, and increasing human activity in urban and peri-urban environments. Climate change has played a significant role in altering the ecology of Ixodidae ticks across Europe, extending their seasonal activity, increasing survival, and enabling populations to expand into new habitats. These patterns are particularly evident in northern and temperate Europe, including the Baltic region, where rising temperatures and milder winters during the last decade have facilitated an earlier seasonal activity and a prolonged duration of questing activity of ixodid ticks, enabling the persistence and spread of a wide range of tick-borne pathogens (TBPs) across diverse climatic regions [1,2].

Lithuania is considered a highly endemic country for tick-borne encephalitis (TBE) and Lyme borreliosis (LB) [3,4]. The climatic conditions in Lithuania are highly suitable and, due to climate change, increasingly favourable for *Ixodes ricinus* tick, the primary vector for these diseases. The country’s temperate climate, characterized by moderate temperatures, high humidity, and sufficient vegetation cover, provides optimal conditions for the entire life cycle of the tick [5,6]. While TBE and LB are the most commonly recognized tick-borne infections in humans across Europe, other infections, including anaplasmosis, neoehrlichiosis, spotted fever group rickettsioses, and babesiosis, are likely underreported due to non-specific clinical symptoms and limited routine diagnostic testing in some regions [7]. This suggests that the true burden of TBPs may be underestimated, particularly in areas with high tick abundance.

Traditionally, the risk of tick-borne infections was primarily associated with natural and semi-natural habitats such as deciduous and mixed forests, forest edges, and meadow ecosystems. However, this paradigm has shifted in recent decades, as urban expansion, landscape fragmentation, and the adaptation of wildlife hosts to anthropogenic environments have reshaped traditional tick habitats and facilitated the establishment of tick populations within cities and peri-urban green spaces [8,9,10]. Recent studies in central and northern Europe show that ticks in urban areas can occur at densities and pathogen prevalence levels comparable to those observed in natural forested habitats [9,11,12,13]. Such patterns are likely driven by the presence of vertebrate host communities and connected habitat networks within urban and peri-urban green spaces, which facilitate the maintenance and spread of *I. ricinus* and associated TBPs between natural and anthropogenic environments [7,8,14,15].

Most previous studies on TBPs in Lithuania have focused on natural habitats, reflecting the long-standing assumption that the highest risk of acquiring TBPs is associated with deciduous and mixed woodlands. The prevalence of *Borrelia burgdorferi* sensu lato in *I. ricinus* has been shown to vary across different habitat types [16]. The highest infection rate was observed in deciduous and mixed forests (20%), lower in pine forests (7.4%), and lowest in agricultural areas (2.4%), reflecting differences in vegetation structure, humidity, and host availability. Although the geographical differences in the prevalence of *B. burgdorferi* s.l. were also detected (varied from 1.1% to 31.9%), no significant correlation with location has been observed, indicating that local ecological factors, rather than region, primarily determine infection risk. These findings highlight the influence of habitat and reservoir host communities on the circulation of *B. burgdorferi* s. l. in Lithuanian tick populations.

Other tick-borne pathogens, including *Anaplasma phagocytophilum*, *Rickettsia* spp., and *Babesia* spp., have also been detected in Lithuania. *Anaplasma phagocytophilum* has been reported in cervids, European bison, and in ticks from wild carnivores [17,18,19], with an overall prevalence of infection in questing *I. ricinus* of 2.9% [19]. Although human granulocytic anaplasmosis has not yet been diagnosed in Lithuania, the presence of infected ticks suggests potential risk, particularly in areas with abundant reservoir hosts such as roe deer (*Capreolus capreolus*) and red deer (*Cervus elaphus*). *Babesia* spp. have been detected in 9.5% of questing ticks, including zoonotic species *Babesia microti* and *Babesia venatorum* [20]. Still, no autochthonous human babesiosis cases have been reported in Lithuania. Spotted fever group *Rickettsia* spp. were detected in 17% of examined *I. ricinus* (ranging from 0% to 31.3% across different natural habitats in Lithuania), with *Rickettsia helvetica* identified as the predominant species [21].

Urban and suburban areas in Lithuania, especially in and around major cities, combine densely built environments with peri-urban green spaces such as parks, public gardens, recreational areas, and riverine corridors that provide favourable microhabitats for ticks and increase opportunities for human exposure. Despite the widespread use of these green spaces, data on TBP prevalence and habitat-specific risk patterns within urbanized landscapes in Lithuania remain limited. This study was conducted in Kaunas County, a region in Central Lithuania characterized by predominantly flat or gently undulating lowland terrain with landscapes dominated by agricultural fields, river basins, and interspersed forest patches, and a relatively mild humid continental climate compared with other regions at similar latitudes. Kaunas County is shaped by the major Nemunas and Neris rivers and includes wetland habitats such as the Kaunas Reservoir, forming a heterogeneous landscape that integrates densely urbanized areas, peri-urban zones, and semi-natural habitats [22,23]. Ticks were collected from frequently visited recreational, leisure, and memorial sites in urban and suburban areas of Kaunas County. The collected ticks were examined for the presence of TBPs, including *Borrelia* spp., *A. phagocytophilum*, *Neoehrlichia mikurensis*, *Rickettsia* spp., and *Babesia* spp. Based on the described context, our study aimed to investigate habitat-specific patterns in tick density and TBPs across urban and suburban landscapes, in order to better assess potential public health risks in these environments.

## 2. Materials and Methods

### 2.1. Study Sites

**Figure 1:**
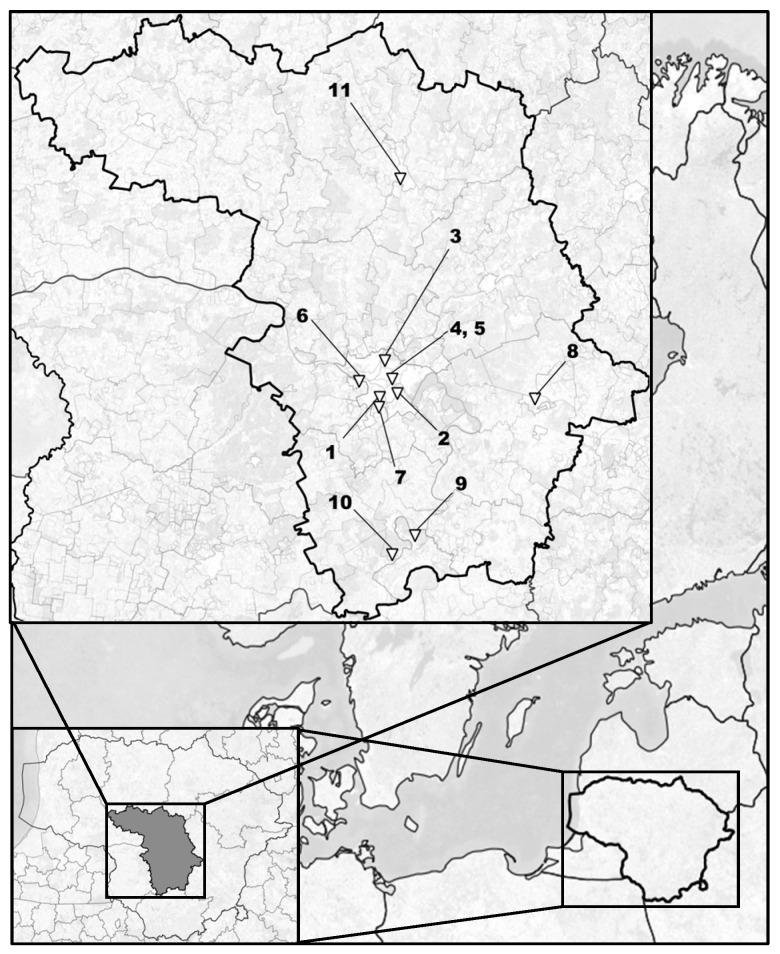
Tick collection sites across Kaunas County, Lithuania: 1—Botanical Garden; 2—Panemunė Forest Park; 3—Kleboniškis Forest Park; 4—Oak Grove Park; 5—Lithuanian Zoological Garden; 6—Academy Campus; 7—III Fort of Kaunas Fortress; 8—Kaišiadorys Forest Park; 9—Birštonas Central Park; 10—Nemunas Loops Regional Park; 11—Kėdainiai cemetery. Map created in QGIS. Administrative boundaries and other vector data derived from OpenStreetMap shapefiles [24]; map data © OpenStreetMap contributors.

Ticks were collected from 11 sites across Kaunas County that are commonly frequented by humans, covering a range of urban (n = 8; sites 1–5, 8, 9, 11) and suburban (n = 3; sites 6, 7, 10) habitats, including urban forest parks, an oak park, a botanical garden, a zoological garden, a national regional park, semi-urban green spaces and a cemetery (Figure 1). Five of these sites were located within Kaunas city (sites 1–5) and its surrounding suburban areas (sites 6,7) (for a detailed description, see Supplementary Material S1).

### 2.2. Tick Collection and Identification

Questing ticks were collected using the standard flagging and dragging methods by one to three persons at each site during the spring of 2021–2025; however, not all sites were sampled each year. At each site, from one to three sampling sessions were conducted. Ticks were identified to the species level, life stage, and sex using taxonomic keys [25,26] and a stereo microscope Motic SMZ-171 (Motic, Hong Kong, China).

### 2.3. DNA Extraction and Pathogen Screening

Genomic DNA from ticks was extracted using 2.5% ammonium hydroxide solution [27]. The samples were screened for the presence of TBPs using real-time PCR assays designed to amplify fragments of specific genes from *Borrelia* spp., *A. phagocytophilum*, *N. mikurensis*, *Rickettsia* spp., and *Babesia* spp. The real-time PCR reactions were performed on Rotor Gene Q (Qiagen, Venlo, The Netherlands) in a total volume of 15 μL, consisting of 1× Sensi Mix™ II Probe No-ROX (Bioline, London, England), 1 pM of each primer, 0.5 pM of each probe, and 1 μL of extracted DNA. The PCR conditions were as follows: an initial denaturation at 95 °C for 10 min, followed by 50 cycles of denaturation at 95 °C for 20 s, annealing at 60 °C for 1 min, and extension at 72 °C for 20 s. Samples with cutoffs below 39 Ct (cycle threshold) and when the threshold was 0.10101 were considered positive.

For identification of pathogen species, amplification of partial gene regions through conventional and nested PCRs, and in some cases, PCR-RFLP, was used. Negative (sterile, double-distilled water instead of DNA) and positive (DNA of pathogen-infected ticks, confirmed by sequencing) controls were included in each PCR run.

The following pathogen groups were analyzed.

#### 2.3.1. *Borrelia* spp.

All samples were screened for *B. burgdorferi* s. l. and *Borrelia miyamotoi* using duplex real-time PCR assays targeting the *23S rRNA* (77 bp) and *fla* (chromosomal flagellin, 156 bp) gene fragments, following previously published protocols [28,29]. Species identification was confirmed using PCR-RFLP with HpyF3I restriction enzyme [30] or gene-specific conventional and nested PCRs targeting the *ospA* (outer surface protein A) and *fla* genes, and 16S-23S *rrs-rrlA* intergenic spacer region (IGS) [31,32,33].

#### 2.3.2. *Anaplasma phagocytophilum* and *Neoehrlichia mikurensis*

Detection of *A. phagocytophilum* and *N. mikurensis* in tick samples was carried out using a duplex real-time PCR assay, targeting the *msp2* (98 bp) and *groEL* (129 bp) gene fragments [34,35]. Samples positive for *A. phagocytophilum* and *N. mikurensis* were further analyzed using a nested PCR assay amplifying partial *groEL* gene sequences, as described by Liz et al. [36].

#### 2.3.3. *Rickettsia* spp.

Detection of spotted fever group *Rickettsia* DNA was performed using a duplex real-time PCR targeting two distinct regions of the *gltA* gene, allowing simultaneous detection of *Rickettsia* spp. (103 bp) and *R. helvetica* (125 bp) [37,38]. All *Rickettsia*-positive tick samples were further analyzed using nested PCR amplification of the 338 bp *gltA* gene fragment [39].

#### 2.3.4. *Babesia* spp.

*Babesia* spp. were detected using a duplex real-time PCR assay designed to amplify two *Babesia* regions: a 104 bp fragment of the internal transcribed spacer region (ITS) and a 62 bp fragment of the *18S rRNA* gene, following protocols adapted from Azagi et al. [40] and Øines et al. [41]. This assay enables sensitive detection of both Clade I (“*B. microti*-like”) and Clade X *Babesia* species circulating in European tick populations. Samples positive for *Babesia* DNA in the real-time PCR were further confirmed using nested PCR targeting the *18S rRNA* gene fragment [42,43].

PCR amplicons from positive samples were excised from 1.5% agarose gels and purified using the GeneJET Gel Extraction Kit (Thermo Fisher Scientific, Vilnius, Lithuania) according to the manufacturer’s instructions. The purified products were sent for sequencing to Macrogen (Amsterdam, The Netherlands).

### 2.4. Statistical Analysis

*Ixodes ricinus* density was calculated using the number of ticks collected per unit of time method [44,45,46,47] as the sum of all adults and nymphs collected, divided by the number of person-hours (number of people × hours spent sampling) of sampling conducted at a given site. For sites with multiple sampling sessions, overall (pooled) tick density was calculated as the total number of ticks divided by the total person-hours across all sessions. Mean session density was calculated as the arithmetic mean of session-specific densities. Confidence intervals (95% CI) for mean session densities were estimated assuming a Poisson distribution of tick counts per person-hour. Due to the non-normal distribution of tick densities and unequal numbers of sessions per site (1–3 per site), a non-parametric statistical method was applied. Differences in tick densities among sites were evaluated using the Kruskal–Wallis test, which compares median values across multiple groups. Analysis was conducted in R (version 4.5.3). A significance threshold of *p* < 0.05 was applied.

Pathogen prevalence was calculated as the percentage (%) of PCR-positive ticks among the total number of examined ticks within each developmental stage. The prevalence of five pathogen groups—*Borrelia* spp., *Babesia* spp., *A. phagocytophilum*, *N. mikurensis*, and *Rickettsia* spp.—was compared between tick life stages (nymphs and adults) and sampling sites. For each pathogen, differences in prevalence between sampling sites and developmental stages were evaluated using the chi-square (χ^2^) test of independence. When the assumptions of the χ^2^ test were not met (i.e., when expected cell counts were <5), Fisher’s exact test was applied. To quantify the strength and direction of associations between developmental stage and pathogen detection, odds ratios (OR) with 95% confidence intervals (95% CI) were calculated. An OR = 1 indicates no difference in infection odds between adults and nymphs; OR > 1 indicates higher odds of infection in adults compared to nymphs; and OR < 1 indicates lower odds of infection in adults relative to nymphs. Statistical significance was determined based on *p*-values and whether the 95% CI excluded the value 1. Descriptive statistical analysis was performed using SPSS software (IBM SPSS Statistics for Windows, version 29.0).

### 2.5. GenBank Accession Numbers

Representative pathogen sequences obtained in this study were deposited in the GenBank database.

*Borrelia afzelii*: PV081830–PV081836, PV081838–PV081841, PV081845–PV081850, PV081854 (*OspA*); PQ473541, PQ473545–PQ473547, PQ473560, PQ473568, PQ473572, PX998194–PX998206, PQ473548 (*FlaB*); PV115019–PV115023, PV115026–PV115027 (ITS). *Borrelia garinii*: PV081787, PV081790, PV081792, PV081794–PV081798, PV081800–PV081806, PV081808 (*OspA*); PQ473575, PQ473585–PQ473588, PX998188–PX998193 (*FlaB*); PV115014–PV115018 (ITS). *Borrelia burgdorferi* sensu stricto: PV081811, PV081813–PV081814 (*OspA*); PX998208 (*FlaB*); PV115007–PV115008 (ITS). *Borrelia miyamotoi*: PQ473606, PQ473610–PQ473611, PX998207 (*FlaB*); PV114994–PV115000 (ITS). *Borrelia lusitaniae*: PQ473598 (*FlaB*). *Borrelia valaisiana*: PX998209 (*FlaB*). *Anaplasma phagocytophilum*: PV711389–PV711401, PX994912 (*groEL*). *Neoehrlichia mikurensis*: PV711403–PV711411 (*groEL*). *Rickettsia helvetica*: PX994913 (*gltA*). *Rickettsia monacensis*: PZ060412 (*gltA*). *Babesia microti*: PZ059349–PZ059353 (*18S rRNA*). *Babesia venatorum*: PZ059354–PZ059359 (*18S rRNA*). *Babesia capreoli*: PZ059360 (*18S rRNA*).

## 3. Results

A total of 1539 *I. ricinus* ticks (474 females, 453 males, and 612 nymphs) over 30.5 person-hours of field sampling were collected from different urban (n = 8) and suburban (n = 3) areas across Kaunas County. The number of ticks collected from each sampling site ranged from 2 to 407 (Table 1). Total location-specific *I. ricinus* density ranged from 0.67 to 135.67 ticks per person-hour.

The prevalence of pathogens in ticks was estimated based on the results of real-time PCR. Of the 1539 *I. ricinus* ticks tested, 39.0% (600) carried at least one pathogen. Among the detected pathogens, *Borrelia* spp. was the most prevalent (24.43%; 95% CI: 22.35–26.64), followed by *Rickettsia* spp. (8.64%; 95% CI: 7.4–10.2) and *N. mikurensis* (8.19%; 95% CI: 6.9–9.7), while *A. phagocytophilum* (3.96%; 95% CI: 3.1–5.1) and *Babesia* spp. (2.53%; 95% CI: 1.9–3.5) were detected less frequently.

The prevalence of each TBP varied considerably between locations. Significant differences in pathogen prevalence between sampling sites were observed for *Borrelia* spp. (χ^2^ = 36.00, *p* < 0.001) and *N. mikurensis* (χ^2^ = 22.29, *p* = 0.014), whereas no statistically significant differences were found for other pathogens.

Overall, the prevalence of *Borrelia* spp. was significantly higher in adult ticks (26.75%, 248/927) than in nymphs (20.92%, 128/612) (χ^2^ = 6.8, *p* = 0.009; 95% CI:1.08–1.76). Similarly, *A. phagocytophilum* was more prevalent in adults (4.96%, 46/927) than in nymphs (2.45%, 15/612) (χ^2^ = 6.11, *p* = 0.013; 95% CI:1.15–3.76). In contrast, no significant differences between life stages were observed for *N. mikurensis* (8.74% vs. 7.35%; χ^2^ = 0.95, *p* = 0.33;95% CI: 0.83–1.77), *Rickettsia* spp. (8.63% vs. 8.66%; χ^2^ = 0.0004, *p* = 0.984; 95% CI:0.69–1.43), or *Babesia* spp. (2.16% vs. 3.1%; χ^2^ = 1.35, *p* = 0.25; 95% CI:0.36–1.30).

Six *Borrelia* species were detected, five from the *B. burgdorferi* s.l. complex and *B. miyamotoi* from the relapsing fever group, along with three *Babesia* species and two *Rickettsia* species at the investigated sampling sites (Table 2).

Co-infections were detected in multiple combinations across the investigated sites (Table 3). The most frequent co-infection was *B. burgdorferi* s. l. and *N. mikurensis* (n = 37), followed by *B. burgdorferi* s.l. and *R. helvetica* (n = 11). Triple and more complex co-infections were rare and occurred only sporadically.

### 3.1. Tick Density Prevalence, Species Diversity, and Co-Infection of TBPs in the Investigated Locations

#### 3.1.1. Botanical Garden (Kaunas)

A total of 93 females, 105 males, and 103 nymphs of *I. ricinus* were collected in the Kaunas Botanical Garden. Overall tick density across three sampling sessions was 75.25 ticks per person-hour. Session-specific densities ranged from 65 to 103 ticks per person-hour. The mean session density was 78.17 ticks per person-hour (95% CI: 66.75–83.75). All of the examined pathogens were detected at this site (Table 1). The prevalence of each pathogen differed by tick developmental stage and sex (Table S1). Four *Borrelia*, two *Rickettsia*, and two *Babesia* species were identified (Table 2). Among the 26 detected co-infections, double co-infections were the most frequent (Table 3).

#### 3.1.2. Panemunė Pinewood Park (Kaunas)

A total of 62 females, 41 males, and 69 nymphs of *I. ricinus* were collected in the Panemunė Pinewood Park. Overall tick density across three sampling sessions was 43 ticks per person-hour. Session-specific densities ranged from 31 to 51 ticks per person-hour. The mean session density was 42.33 ticks per person-hour (95% CI: 36.57–49.43). All of the examined pathogens were detected at this site (Table 1). The prevalence of each pathogen differed by tick developmental stage and sex (Table S1). Four *Borrelia* species, *B. venatorum*, and *R. helvetica* were identified (Table 2). A total of seven co-infections were detected, most commonly involving *Borrelia* spp. and *N. mikurensis* (Table 3).

#### 3.1.3. Kleboniškis Forest Park (Kaunas)

A total of 89 females, 75 males, and 243 nymphs of *I. ricinus* were collected in Kleboniškis Forest Park. Overall tick density across three sampling sessions was 135.67 ticks per person-hour. Session-specific densities ranged from 59 to 202 ticks per person-hour. The mean session density was 135.67 ticks per person-hour (95% CI: 122.49–148.85). All of the examined pathogens were detected at this site (Table 1). The prevalence of each pathogen differed by tick developmental stage and sex (Table S1). Four *Borrelia* species, three *Babesia* species, and *R. helvetica* were identified (Table 2), with 46 cases of co-infections detected (Table 3).

#### 3.1.4. Oak Grove Park and Zoological Garden (Kaunas)

In Oak Grove Park, where three sampling sessions were conducted, only two specimens of *I. ricinus* males were collected. The overall tick density across the three sessions was 0.67 ticks per person-hour, and the mean session density was also 0.67 ticks per person-hour. None of the tested pathogens were detected in ticks from this location.

In the Zoological Garden, a total of 24 females, 22 males, and 14 nymphs of *I. ricinus* were collected during two sampling sessions. The overall tick density was 8.57 ticks per person-hour, with session-specific densities ranging from eight to nine ticks per person-hour. The mean session density was 8.5 ticks per person-hour (95% CI: 6.40–10.74). Three of the examined pathogens were detected at this site (Table 1). Prevalence of *Borrelia* spp. was similar across all life stages, while *N. mikurensis* and *Rickettsia* spp. varied by developmental stage and sex. *Anaplasma phagocytophilum* was identified in one male tick (Table S1). Three *Borrelia* species and *R. helvetica* were identified (Table 2). No co-infections were observed.

#### 3.1.5. Academy Campus (Kaunas District)

A total of 19 females, 26 males, and 11 nymphs of *I. ricinus* were collected at the Academy Campus sampling site. Tick density was 28.0 ticks per person-hour.

Four of the examined pathogens were detected at this site (Table 1). The prevalence of each pathogen differed by tick developmental stage and sex (Table S1). Three *Borrelia* species, two *Babesia* species, and *R. helvetica* were identified (Table 2). Two co-infection cases were observed (Table 3).

#### 3.1.6. III Fort of Kaunas Fortress (Kaunas District)

A total of 2 females, 11 males, and 13 nymphs of *I. ricinus* were collected from vegetation across the III Fort of Kaunas Fortress. Tick density was 26.0 ticks per person-hour. Pathogens from two genera were detected at this site (Table 1), with prevalence varying by tick developmental stage and sex (Table S1). Two pathogen species were identified: *B. afzelii* and *R. helvetica* (Table 2). One co-infection was recorded (Table 3).

#### 3.1.7. Kaišiadorys Forest Park (Kaišiadorys)

A total of 19 females, 24 males, and 76 nymphs of *I. ricinus* were collected from Kaišiadorys Forest Park. Tick density was 119 ticks per person-hour. All of the examined pathogens were detected at this site (Table 1). The prevalence of each pathogen differed by tick developmental stage and sex (Table S1). Three *Borrelia* species and *R. helvetica* were identified (Table 2). No co-infections were observed at this site.

#### 3.1.8. Central Park (Birštonas)

A total of 97 females, 86 males, and 13 nymphs of *I. ricinus* were collected from Central Park in Birštonas during two sampling sessions. The overall tick density was 130.67 ticks per person-hour, with session-specific densities ranging from 44 to 174 ticks per person-hour. The mean session density was 109 ticks per person-hour (95% CI: 112.37–148.96). All examined pathogens were detected at this site (Table 1) and were found exclusively in adult ticks (Table S1). Four *Borrelia* species, *B. venatorum*, and *R. helvetica* were identified (Table 2). In total, 19 co-infections were recorded (Table 3).

#### 3.1.9. Nemunas Loops Regional Park (Prienai District)

A total of 41 females, 37 males, and 68 nymphs of *I. ricinus* were collected from the sampling site located in Nemunas Loops Regional Park during two sampling sessions. The overall tick density was 48.67 ticks per person-hour, with session-specific densities ranging from 47 to 49.5 ticks per person-hour. The mean session density was 48.25 ticks per person-hour (95% CI: 40.77–56.56). All of the examined pathogens were detected at this site (Table 1). The prevalence of each pathogen differed by tick developmental stage and sex (Table S1). Three *Borrelia* species, two *Babesia* species, and *R. helvetica* were identified (Table 2). Six co-infections were recorded (Table 3).

#### 3.1.10. Cemetery (Kėdainiai)

A total of 28 females, 24 males, and two nymphs of *I. ricinus* were collected from the green area of the cemetery in Kėdainiai. Tick density was 54 ticks per person-hour. Three of the examined pathogens were detected at this site (Table 1). *Borrelia* spp., *N. mikurensis*, *Anaplasma phagocytophilum,* and *Babesia* spp. were detected only in adult ticks, with prevalences varying between males and females, while *Rickettsia* was detected in one male and was the only pathogen identified in nymphs (Table S1). Two *Borrelia* species and *R. helvetica* were identified (Table 2). Six cases of dual co-infection were detected (Table 3).

## 4. Discussion

Over the past century, Lithuanian cities have undergone substantial transformations in their urban structure. Historically, compact towns have gradually evolved into large cities. Increasing anthropogenization has substantially altered the natural environment. Urban expansion has reduced the extent of natural areas and increased the vulnerability and fragmentation of ecological components [22,48]. In Kaunas County, ongoing urban development has modified landscape structure and connectivity, although surrounding forests help regulate microclimatic conditions by influencing air humidity, temperature, and wind exposure, thereby contributing to a relatively mild regional microclimate [18]. Kaunas, the second-largest city, remains one of the greenest in Lithuania. Situated at the confluence of the Nemunas and Neris rivers, the city is characterized by a dense network of river valleys, slopes, and green corridors that penetrate the urban matrix. The landscape structure of Kaunas represents a mosaic of natural and anthropogenic elements [22]. In Kaunas, there are an oak park and forest parks, a zoo, and a botanical garden. This heterogeneous landscape provides suitable habitats for wildlife hosts, supporting tick populations and the circulation of tick-borne pathogens across urban and suburban environments.

This study investigated a gradient of green spaces, from large, semi-natural or contiguous forested habitats (Kleboniškis Forest Park, Panemunė Pinewood Park, Nemunas Loops Regional Park), to ecologically complex mosaic habitats (Birštonas Central Park, Kaišiadorys Forest Park, VMU Botanical Garden), and to intensively managed urban or suburban areas (Kaunas Fortress III Fort, Academy Campus, Oak Grove Park, the Lithuanian Zoological Garden, and Kėdainiai Cemetery).

### 4.1. Habitat Structure and Tick Density

Tick densities varied considerably among the study sites, ranging from 0.67 ticks per person-hour in Oak Grove Park to 135.67 ticks per person-hour in Kleboniškis Forest Park.

The highest tick densities were recorded in Kleboniškis Forest Park and Kaišiadorys Forest Park. Both sites retain characteristics of semi-natural or minimally disturbed woodland ecosystems. Although located near the city centre, Kleboniškis Forest Park retains the characteristics of a natural mixed forest. Restricted access to inner forest areas and partial natural isolation by the Neris River may further support stable wildlife host communities and create optimal conditions for tick survival and reproduction. Similarly, Kaišiadorys Forest Park combines managed recreational zones with less disturbed forest sections, creating heterogeneous microhabitats with sufficient humidity and abundant wildlife hosts. Dense forest structure, shaded understory, and ecological continuity likely promote high tick survival and successful completion of the tick life cycle. Birštonas Central Park also showed high tick densities. Although managed for recreation, its proximity to surrounding pine forests and riverine habitats likely facilitates wildlife movement, particularly of roe deer and small mammals, sustaining tick populations. The interface between lawns and adjacent forest creates favourable ecotones that promote tick survival and host contact. Previous studies indicate that landscape connectivity and ecotonal interfaces between managed parkland and adjacent woodland can enhance host movement and increase tick abundance [9,49]. Relatively high tick density was observed in the botanical garden, an ecologically complex yet managed urban site. Despite regular maintenance and high visitor numbers, the garden’s heterogeneous vegetation structure, pond system, and diverse plant collections create favourable microclimatic conditions for off-host tick survival and development, while also supporting a wide range of vertebrate hosts that provide blood meals for different tick life stages. In addition, occasional access by larger wildlife, such as roe deer through fence gaps, together with high bird diversity, may further facilitate tick introduction, dispersal, and long-term maintenance of local *I. ricinus* populations. Tick densities at the site in the Nemunas Loops Regional Park near Paduoblis village, the Kėdainiai cemetery site, the Academy campus, and the III Fort likely reflect the mosaic landscape structure. Ecotonal zones between forest patches and anthropogenic areas are often associated with moderate tick densities due to the coexistence of wildlife hosts and human disturbance. However, habitat fragmentation and frequent human activity may reduce host abundance or disrupt tick–host interactions, thereby limiting population growth compared with large, contiguous forests [8,9]. Panemunė Pinewood Park, despite being located within a structurally diverse mixed forest, exhibited lower tick density compared with other investigated forest parks. Intensive recreational use, well-developed infrastructure, and sustained human activity may disrupt tick–host interactions and alter wildlife movement patterns in heavily visited areas. Although mature pine and oak stands provide structural heterogeneity, elevated anthropogenic pressure likely reduces habitat suitability and moderates overall *I. ricinus* abundance. The lowest tick densities were observed in Oak Grove Park and the Lithuanian Zoological Garden. Oak Grove Park is characterized by open, regularly mowed areas with limited understory and reduced humidity—conditions generally unfavourable for tick survival. Moreover, its isolation within dense urban infrastructure likely restricts the movement of large wild hosts, such as roe deer, which are essential for sustaining adult tick populations. Although the Zoological Garden hosts numerous captive animal species, ticks were collected outside the enclosures, where vegetation is intensively maintained and spatially fragmented. Zoological gardens are popular urban recreational areas with a semi-forested or park-like character. The seminatural, fragmented environments characteristic of zoos are designed to host various animal species with differing habitat requirements, which can positively influence the life cycle of ticks. Consequently, the animal species kept in zoos can potentially serve as reservoirs for ticks and TBPs [50]. Despite these localized host resources, the overall ecological conditions in the surrounding vegetation appear less suitable for sustaining high *I. ricinus* densities.

Overall, tick density was highest in large, structurally complex forested habitats and ecotonal environments, moderate in mixed or semi-natural peri-urban landscapes, and lowest in intensively managed or highly modified urban green spaces. This pattern reflects the combined influence of vegetation complexity, microclimatic stability, host availability, and landscape connectivity on *I. ricinus* populations. Even small differences in habitat structure and wildlife presence can therefore result in substantial variation in tick abundance across urban and suburban environments [51,52]. However, despite the apparent differences in mean tick densities, a Kruskal–Wallis test indicated no statistically significant differences between sites (χ^2^ = 12.495, df = 10, *p* = 0.2533), likely due to high within-site variability.

### 4.2. Habitat Structure and TBPs Prevalence

TBPs prevalence differed among the investigated areas. *Borrelia* spp. were the dominant pathogens and were detected at all sampling sites, with prevalence ranging from 13.37% to 38.89%, indicating their strong ecological adaptability in both urban and suburban environments. Their occurrence in both forested and managed habitats suggests a broad ecological tolerance and the ability to persist under diverse environmental conditions. The predominance of *Borrelia* in adult ticks, particularly females, likely reflects cumulative pathogen acquisition during successive blood meals.

At least nine genospecies of *B. burgdorferi* s.l. with differing pathogenicities, clinical signs, and reservoir host associations are known to be circulating in Europe [53]. In this study, six *Borrelia* species were detected, five of which belong to the *B. burgdorferi* s.l. complex: *B. afzelii* (detected in 10 sites), *B. garinii* (7 sites), *B. burgdorferi* s.s. (3 sites), *B. valaisiana* (1 site), and *B. lusitaniae* (1 site), as well as *B. miyamotoi* from the relapsing fever complex (7 sites) (Table 2). The widespread circulation of *B. afzelii* and *B. miyamotoi* suggests a prominent role of rodent reservoirs, whereas the presence of *B. garinii*, *B. valaisiana*, and *B. lusitaniae* indicates involvement of birds and lizards [54,55]. *Borrelia burgdorferi* s.s. is considered a generalist species associated with different vertebrate species such as rodents, hedgehogs, or birds, suggesting these hosts contribute to its persistence in the studied habitats [56]. In most sites, three to four *Borrelia* species were detected, whereas only the cemetery and III Fort harboured one or two species, respectively. However, true species diversity may be underestimated because not all PCR-positive samples from each site were successfully sequenced to identify the specific *Borrelia* species.

*Rickettsia* spp. were detected at all sites, with prevalence ranging from 3.70% to 14.52%. *Rickettsia helvetica* was identified in 99% of positive samples. Similar or higher prevalence rates (9–18.7%) have been reported in other European countries, where *R. helvetica* was likewise the predominant species in urban and peri-urban landscapes [12,57,58,59]. The widespread occurrence of *R. helvetica* and its relatively stable prevalence across habitat types may be explained by its ability to persist in tick populations through both transstadial and transovarial transmission, allowing *I. ricinus* to serve as both a vector and a reservoir host [60]. Detection in both nymphs and adults indicates a stable local circulation, largely independent of the intensity of habitat disturbance.

In contrast, *N. mikurensis* and *A. phagocytophilum* showed more host-dependent patterns. *Neoehrlichia mikurensis* was widely distributed across most study sites, with prevalence ranging from 3.49% to 16.67%. Lower prevalence was observed in semi-natural forested areas, while no infected ticks were found in some intensively managed sites (Table 1). This pattern suggests that *N. mikurensis* circulation is not restricted to forested areas but is closely associated with habitats supporting stable rodent populations, including fragmented urban and suburban green spaces. Similar findings have been reported across Europe, where prevalence in questing *I. ricinus* typically ranges from 1 to 11% in urban, suburban, and sylvatic habitats, with focal areas reaching more than 20% [9]. Lower prevalence has often been documented in highly urbanized Central European sites (e.g., Slovakia, Poland, Czech Republic), whereas higher rates were reported in certain natural or suburban habitats in Germany, the Netherlands, Belgium, and Italy, indicating strong local ecological influence [9].

In our study, *A. phagocytophilum* showed a lower and more homogeneous distribution among the investigated locations, with an overall prevalence of 3.96%. Site-specific prevalence in our study ranged from 0.84% to 5.16% (Table 1). By comparison, studies conducted in neighbouring countries, Poland, Latvia, and Estonia, have reported significantly lower prevalence rates, at 1.7%, 1.1%, and 0.5%, respectively [12,61,62]. *Anaplasma phagocytophilum* appeared more habitat-dependent, with higher infection rates detected in forested areas, where large mammals such as roe deer are present, supporting the importance of large ungulates in maintaining this pathogen. These findings are consistent with other European studies reporting relatively high infection rates (ranging from 2.9% to 14.5%) in urban and suburban *I. ricinus* populations, where diverse mammalian hosts, including ungulates, are present [9].

*Babesia* spp. showed the lowest overall prevalence among the investigated pathogens and were detected in 63.6% study sites. Their occurrence was mainly associated with forested habitats and ecotone areas. Across Europe, *Babesia* prevalence in questing *I. ricinus* from urban and suburban habitats generally ranges from 0.2% to 5%, with occasional higher focal values in natural forest ecosystems, consistent with the patterns observed in our study [51,61,63,64,65]. *Babesia* spp. were not detected in intensively managed urban and suburban locations, including Oak Grove Park, the Zoological Garden, the Academy Campus, and the III Fort (Table 1). Species identification revealed the presence of *B. venatorum*, *B. microti*, and *B. capreoli* (Table 2). However, species diversity may be underestimated, as not all PCR-positive samples were successfully sequenced. *B. venatorum* was the most widely distributed species, occurring in both semi-natural forests and mosaic urban habitats, suggesting ecological flexibility and association with cervids, the principal reservoir hosts (Table 2). *Babesia capreoli* was detected only in Kleboniškis Forest Park, consistent with its association with roe deer, which is considered the primary, typically asymptomatic, reservoir host for this *Babesia* species in Europe and more natural woodland ecosystems [9,65]. *Babesia microti*, associated with small rodents [66], was found mainly in structurally heterogeneous habitats that support abundant rodent communities. Overall, the distribution pattern indicates that *Babesia* spp. are more closely linked to habitats supporting stable populations of specific vertebrate hosts, particularly cervids and small mammals, rather than to urban–rural gradients alone. Semi-natural forests and ecotonal mosaic landscapes appear to provide the most favourable ecological conditions for maintaining diverse *Babesia* species.

Co-infections in ticks reflect the complex interactions between multiple pathogens, their vectors, and host communities, and are increasingly recognized as important for understanding tick-borne disease risk. In our study, the overall co-infection rate was 7.3% (113/1539; 95% CI: 6.0–8.6), with 18.8% (113/600; 95% CI: 15.7–22.0) among infected ticks. Co-infections were detected in 72.7% of study sites and varied across habitat types, with higher rates observed in locations characterized by high tick density and diverse host communities. The most common co-infection pattern involved *B. burgdorferi* s.l. combination with *N. mikurensis* or *R. helvetica*, while more complex co-infections involving three or more pathogens were also identified, indicating a complex pathogen circulation network. Such combinations are considered common because these pathogens share the same reservoir hosts, small rodents [57]. Ticks feeding on the blood of an infected rodent can acquire multiple pathogens simultaneously, thereby increasing the likelihood of co-infections [57]. Our findings align with broader European patterns, indicating that forested, lightly managed, or semi-natural habitats support the greatest diversity of tick infections and co-infections. High rodent and bird diversity, abundant forest floor cover, and denser vegetation significantly increase both tick density and the circulation of multiple pathogens. Similar results were reported by [57] in Switzerland, where 20% of *I. ricinus* collected in urban forests and forest parks carried two or three different pathogens. Similarly, in Romania, a high prevalence of mixed infections in urban habitats was detected [67]. Co-infections occurred in 34.3% of infected *I. ricinus* and 69% of engorged ticks collected from hosts, most commonly involving dual infections with combinations of *Borrelia* spp., *Rickettsia* spp., and *A. phagocytophilum*. Co-infections were also detected in rodent and bird tissues. These findings indicate that in urban habitats, multiple pathogens circulate simultaneously and are transmitted through both vectors and reservoir hosts, potentially increasing the risk of human infection [67]. Humans and animals may be exposed to multiple pathogens from a single co-infected tick, depending on the prevalence of pathogens in ticks and hosts, transmission efficiency, and the duration of tick feeding [68].

In our study, the Kėdainiai cemetery showed the highest prevalence of *Borrelia* spp., *N. mikurensis*, and *Babesia* spp. Cemeteries provide favourable habitats for ticks due to abundant small mammals, birds that transport ticks, dense litter, shade, and a humid microclimate. Several previous European studies have shown that cemeteries can be important foci of tick-borne pathogens and, like other urbanized green spaces, support a significant proportion of co-infections, reflecting the intense circulation of multiple pathogens within the diverse wild host community [57,67,69].

From a public health perspective, the widespread detection of medically relevant pathogens across parks, campuses, cemeteries, and historical sites underscores the need for habitat-specific risk assessment. Although urban green spaces provide substantial ecological and social benefits, their design and maintenance should consider microhabitat features that could favour tick survival. Vegetation management, host population monitoring, and public awareness measures may help mitigate exposure risk while preserving urban biodiversity.

## 5. Conclusions

Our findings demonstrate that urban and suburban landscapes are not epidemiologically uniform. Tick-borne pathogen circulation is shaped by local habitat features, host communities, and management intensity. Even small or fragmented green areas can sustain complex enzootic cycles when ecological conditions are suitable. These results highlight the importance of habitat-specific risk assessments and integrated surveillance strategies within urban planning and public health frameworks.

## Figures and Tables

**Table 1:** Overall and location-specific prevalence of tick-borne pathogens in *Ixodes ricinus* ticks.

Location (No)	N	*Borrelia* spp.	*A. phagocytophilum*	*N. mikurensis*	*Rickettsia* spp.	*Babesia* spp.
		n (%)	n (%)	n (%)	n (%)	n (%)
Botanical Garden (1)	301	86 (28.57)	13 (4.32)	31 (10.30)	34 (11.30)	3 (1.00)
Panemunė Pinewood Park (2)	172	23 (13.37)	6 (3.49)	6 (3.49)	10 (5.81)	6 (3.49)
Kleboniškis Forest Park (3)	407	118 (28.99)	21 (5.16)	42 (10.32)	32 (7.86)	14 (3.44)
Oak Grove Park (4)	2	0	0	0	0	0
Zoological Garden (5)	60	13 (21.67)	1 (1.67)	6 (10.00)	9 (15.00)	0
Academy Campus (6)	56	10 (17.86)	1 (1.79)	6 (10.71)	8 (14.29)	0
III Fort of Kaunas Fortress (7)	26	6 (23.08)	0	0	3 (11.54)	0
Kaišiadorys forest park (8)	119	26 (21.85)	1 (0.84)	5 (4.20)	6 (5.04)	4 (3.36)
Central Park (9)	196	52 (26.53)	9 (4.59)	11 (5.61)	19 (9.69)	4 (2.04)
Nemunas Loops Regional Park (10)	146	21 (14.38)	7 (4.79)	10 (6.85)	10 (6.85)	5 (3.42)
Cemetery (11)	54	21 (38.89)	2 (3.70)	9 (16.67)	2 (3.70)	3 (5.56)
Total	1539	376 (24.43)	61 (3.96)	126 (8.19)	133 (8.64)	39 (2.53)

No—number assigned to the sampling site; N—number of collected ticks; n—number of infected ticks, %—prevalence.

**Table 2:** Diversity of tick-borne pathogens in different sampling sites.

Location (No)	*Borrelia*	*Babesia*	*Rickettsia*	*A. phagocytophilum*	*N. mikurensis*
Botanical Garden (1)	*B. burgdorferi* s.l. *, *B*. *burgdorferi* s. s., *B*. *afzelii*, *B*. *garinii*, *B*. *miyamotoi*	*Babesia* spp. *, *B*. *venatorum*, *B*. *microti*	*R*. *helvetica, R*. *monacensis*	+	+
Panemunė Pinewood Park (2)	*B. burgdorferi* s.l. *, *B*. *burgdorferi* s. s., *B*. *afzelii*, *B*. *garinii*, *B. miyamotoi*	*Babesia* spp. *, *B*. *venatorum*	*R*. *helvetica*	+	+
Kleboniškis Forest Park (3)	*B. burgdorferi* s.l. *, *B*. *burgdorferi* s. s., *B*. *afzelii*, *B*. *garinii*, *B*. *miyamotoi*	*Babesia* spp. *, *B*. *venatorum*, *B*. *capreoli*, *B*. *microti*	*R*. *helvetica*	+	+
Zoological Garden (5)	*B. burgdorferi* s.l. *, *B*. *garinii*, *B*. *afzelii*, *B*. *miyamotoi*		*R. helvetica*	+	+
Akademy Campus (6)	*B. burgdorferi* s.l. *, *B*. *afzelii*, *B*. *garinii*, *B*. *miyamotoi*	*Babesia* spp. *, *B*. *venatorum*, *B*. *microti*	*R*. *helvetica*	+	+
III Fort of Kaunas Fortress (7)	*B. burgdorferi* s.l. *, *B*. *burgdorferi* s. l., *B*. *afzelii*		*R*. *helvetica*	+	-
Kaišiadorys Forest Park (8)	*B. burgdorferi* s.l. *, *B*. *afzelii*, *B. lusitaniae, B*. *miyamotoi*		*R*. *helvetica*	+	+
Central Park (9)	*B. burgdorferi* s.l. *, *B*. *afzelii*, *B*. *garinii*, *B*. *valaisiana, B*. *miyamotoi*	*Babesia* spp. *, *B*. *venatorum*	*R*. *helvetica*	+	+
Nemunas Loops Regional Park (10)	*B. burgdorferi* s.l. *, *B*. *afzelii*, *B*. *garinii*, *B*. *miyamotoi*	*Babesia* spp. *, *B*. *venatorum*, *B*. *microti*	*R*. *helvetica*	+	+
Cemetery (11)	*B. burgdorferi* s.l. *, *B*. *afzelii*	*Babesia* spp. *, *B*. *venatorum*	*R*. *helvetica*	+	+

* Pathogens were detected by real-time PCR, but were not identified to the species level by sequence analysis; “+” indicates the detection of pathogens.

**Table 3:** Patterns and distribution of co-infections across study sites.

Co-Infection Combinations	Location (No)										Total
	1	2	3	5	6	7	8	9	10	11	
B. s. l. + N. mik.	11	1	18					4	1	2	37
B. s. l. + R. hel.	3	1	2			1		3	1		11
B. miy. + N. mik.	2		4					1			7
B. afz. + N. mik.		2	3							2	7
B. afz. + B. miy.	1		3		2						6
B. miy. + R. hel.	1		2					1			4
B. s. l. + B. miy.	1	1	1					1			4
B. s. l. + B. miy. + N. mik.	1		3								4
A. ph. + R. hel.	2							1			3
N. mik. + R. hel.	2									1	3
A. ph. + N. mik.			2						1		3
B. s. l. + A. ph.								2		1	3
B. afz. + A. ph.								1	2		3
B. afz. + R. hel.			1					1			2
B. gar. + R. hel.			1					1			2
B. s. l. + B. spp.		1						1			2
B. s. l. + A. ph. + R. hel.	1							1			2
N. mik. + Ba. spp.	1										1
B. afz. + Ba. mic.			1								1
B. miy. + A. ph.								1			1
N. mik. + Ba. ven.		1									1
B. afz. + B. miy. + N. mik.			1								1
B. miy. + N. mik. + R. hel.			1								1
B. afz. + N. mik. + Ba. ven.			1								1
B. s. l. + A. ph. + N. mik. + R. hel.			1								1
B. s. l. + B. miy. + N. mik. + R. hel.									1		1
B. miy. + B. s. l. + N. mik. + R. hel. + Ba. spp.			1								1
Total	26	7	46	0	2	1	0	19	6	6	113

No—number assigned to the sampling site; 1—Botanical Garden; 2—Panemunė Pinewood Park; 3—Kleboniškis Forest Park; 5—Zoological garden; 6—Academy Campus; 7—III Fort of Kaunas Fortress; 8—Kaišiadorys Forest Park; 9—Central Park; 10—Nemunas Loops Regional Park; 11—Cemetery. Abbreviations: B. s. l.—*B. burgdorferi* s.l.; B. afz.—*B. afzelii*; B. gar.—*B. garinii*; B. miy.—*B. miyamotoi*; Ba. mic.—*B. microti*; Ba. spp.—*Babesia* spp.; Ba. ven.—*B. venatorum*; A. ph.—*A. phagocytophilum*; N. mik.—*N. mikurensis*; R. hel.—*R. helvetica*.

## Data Availability

Data is contained within the article.

## Supplementary Materials

The following supporting information can be downloaded at: https://www.mdpi.com/article/10.3390/pathogens15040376/s1, Material S1: Detailed descriptions of the study sites.; Table S1: Location-specific prevalence of tick-borne pathogens in adults and nymphs of *Ixodes ricinus*.
