# Supplementary material for: Habitat-Specific Patterns of Tick-Borne Pathogens in Urban and Suburban Landscapes
Source: Pathogens. 2026 Apr 1;15(4):376. doi: 10.3390/pathogens15040376 (PMC13119425; doi:10.3390/pathogens15040376)
Supplement: Supplementary file 1 [file pathogens-15-00376-s001.zip › pathogens-4209368-Supplementary Material S1.pdf]

Supplementary Material S1. Detailed descriptions of the study sites.

Sampling site 1 - Botanical Garden (Kaunas)

The Vytautas Magnus University (VMU) Botanical Garden is located near the center of Kaunas and covers 62.5 hectares (Figure 1). The territory comprises botanical collections, a greenhouse complex, a large landscaped park composed of mixed broadleaved and coniferous tree stands interspersed with open grass areas and a pond system. The park is managed, with vegetation maintained through regular grass mowing. Despite being fenced, the area remains accessible to wildlife, including roe deer, and supports abundant small mammal communities, particularly rodents, which serve as important hosts for immature tick stages. High diversity of birds may further facilitate tick introduction and dispersal, supporting tick persistence in this urbanized environment. Ticks were collected from vegetation near the pathways and picnic areas.

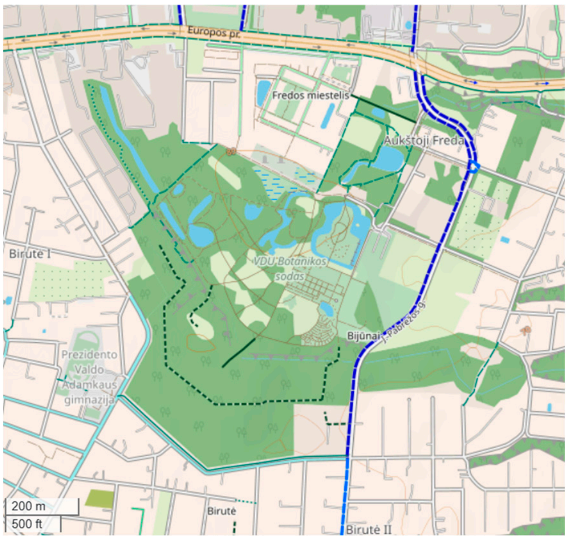

Figure 1. VMU Botanical Garden. Screenshot from OpenStreetMap [24]; map data © OpenStreetMap contributors.

Sampling site 2 - Panemunė Pinewood Park (Kaunas)

Panemunė Pinewood, a large urban forest park, located on the right bank of the Nemunas River, was created in a natural forest and serves as an important urban recreational area (Figure 2). It is dominated by mature *Pinus sylvestris* stands interspersed with old *Quercus robur* trees, forming a structurally diverse mixed forest with sandy soils and dense understory vegetation. The park attracts substantial human activity during the warm season due to extensive recreational infrastructure, such as paved cycling and walking paths, a river beach, playgrounds, fitness facilities, and open spaces for sports and leisure. As part of the Nemunas River woodland ecosystem, the park supports diverse bird communities, as well as typical forest-dwelling mammals, including red squirrels, rodents, hedgehogs, and red foxes. Ticks were collected from vegetation near the pathways and picnic areas.

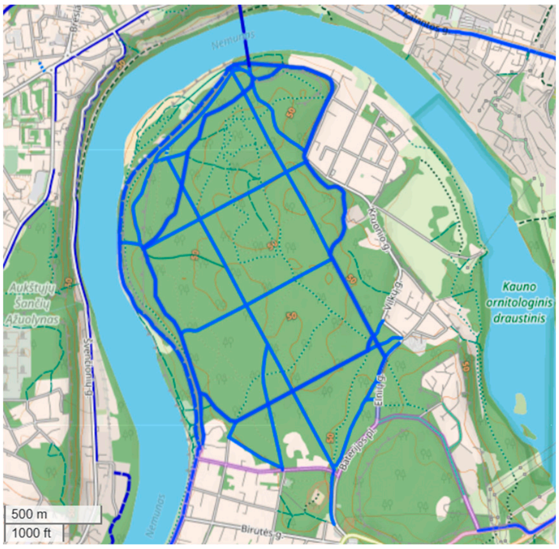

Figure 2. Panemunė Pinewood Park. Screenshot from OpenStreetMap [24]; map data © OpenStreetMap contributors.

Sampling site 3 - Kleboniškis Forest Park (Kaunas)

Kleboniškis Forest Park is one of the largest parks in Kaunas (Figure 3). Despite its proximity to the city center, Kleboniškis Forest retains characteristics of unmanaged, semi-natural woodlands. It is partially isolated by the Neris River, which encircles the area from the west and north, limiting access and reducing anthropogenic disturbance. Human activity is largely concentrated in the southern sector, while the inner and northern parts of the forest experience minimal visitor pressure and thus maintain the appearance of untouched, wild nature. Ticks were collected from vegetation near the pathways and picnic areas.

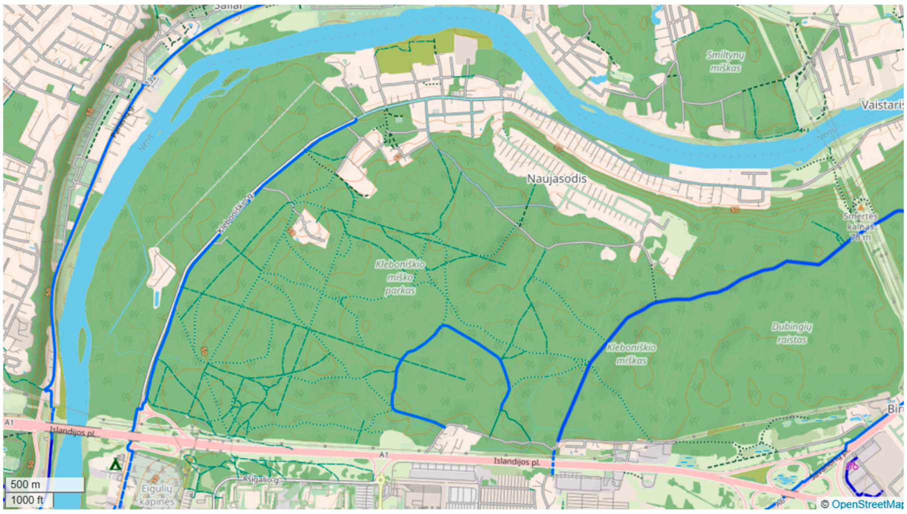

Figure 3. Kleboniškis Forest Park. Screenshot from OpenStreetMap [24]; map data © OpenStreetMap contributors.

Sampling site 4 - Oak Grove Park and site 5 - Lithuanian Zoological Garden (Kaunas)

Oak Grove Park is one of the most distinctive urban green spaces in Kaunas, covering 84.42 ha and representing the largest urban stand of mature oaks in Europe (Figure 4). The park is a popular recreational area with well-maintained paths, sports fields and open spaces. Due to regular mowing and minimal underbrush, the habitat is relatively open, creating less favourable microclimatic conditions for ticks than in denser urban forests. The oak grove is surrounded by urban infrastructure, limiting the movement of large wild mammals such as roe deer, key hosts for adult ticks, thus shaping the park's host community. Ticks were collected along pathways, grassy patches, and ecotone zones within the park, where small mammals and birds may contribute to the maintenance of local tick populations.

The Lithuanian Zoological Garden is located in Oak Grove Park territory and occupies 15.66 ha (Figure 4). The zoo contains numerous enclosures housing a wide variety of mammals and birds, providing localized hotspots of potential hosts and suitable microhabitats for ticks. Ticks were collected outside animal enclosures, where vegetation structure and host availability often create favourable conditions for questing ticks.

The combined oak forest and zoo environment forms a mosaic of contrasting microhabitats, ranging from an intensively maintained recreational area to an animal-associated zone with high host presence.

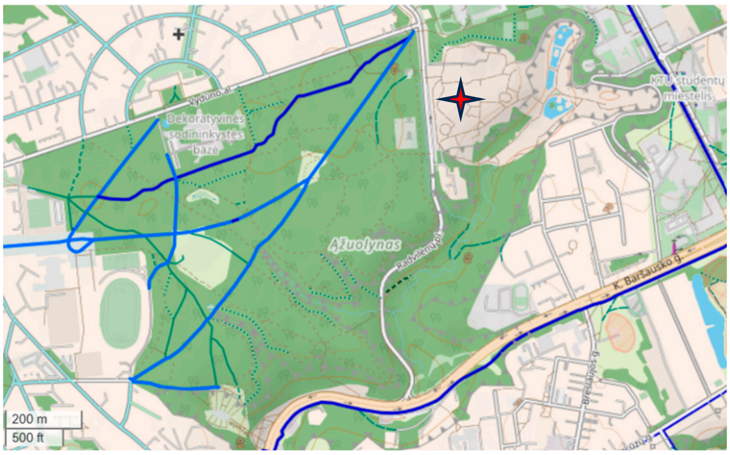

Figure 4. Oak Grove Park and Lithuanian Zoological Garden (marked). Screenshot from OpenStreetMap [24]; map data © OpenStreetMap contributors.

Sampling site 6 - Academy campus (Akademija, Kaunas district)

The Academy campus, located in a suburban area of Kaunas, represents a semi-urban green space characterized by mixed vegetation, open lawns, and patches of shrubs and young woodland (Figure 5). The area is surrounded by student dormitories, academic buildings, a stadium, and lightly trafficked roads, creating a mosaic of anthropogenic and natural elements. Frequent human activity occurs throughout the campus, making it relevant for assessing tick presence and potential circulation of TBPs in a suburban environment. Ticks were collected in the area surrounding the stadium, in the meadow-forest ecotone.

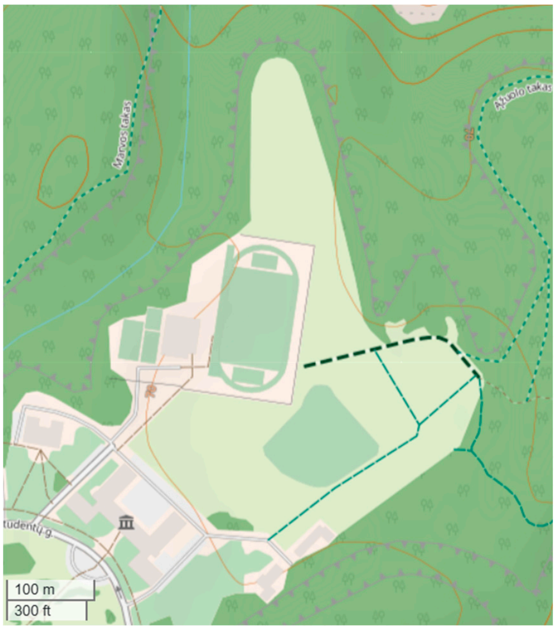

Figure 5. Academy campus. Screenshot from OpenStreetMap [24]; Map data © OpenStreetMap contributors.

Sampling site 7 - Third Fort of the Kaunas Fortress (Seniava, Kaunas district)

The III Fort of the Kaunas Fortress, located in a suburban area of Kaunas, is embedded within residential surroundings (Figure 6). Although anthropogenic influence is strong, patches of unmanaged vegetation, old masonry, and shaded microhabitats around the ramparts provide suitable conditions for tick presence.

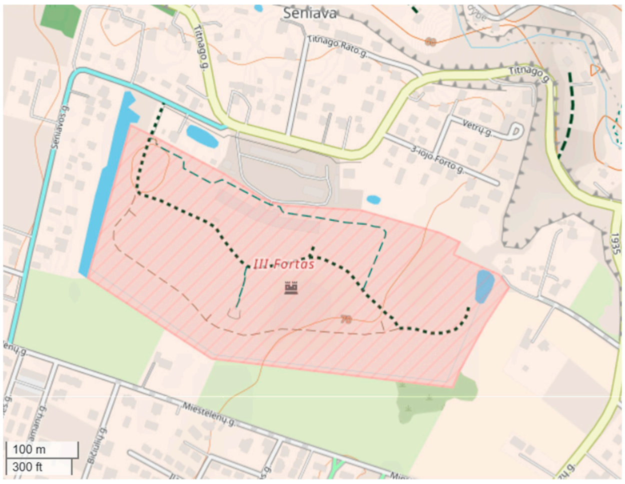

Figure 6. The Third Fort of the Kaunas Fortress. Screenshot from OpenStreetMap [24]; map data © OpenStreetMap contributors.

Sampling site 8 - Kaišiadorys Forest Park (Kaišiadorys)

Kaišiadorys Forest Park, located within the town of Kaišiadorys (Figure 7), consists of mixed woodland with patches of coniferous and deciduous trees, interspersed with walking paths and recreational areas. The combination of managed recreational zones and less disturbed forest sections provides diverse microhabitats suitable for ticks, while frequent human activity creates opportunities for human–tick contact. Ticks were collected along walking paths and picnic areas.

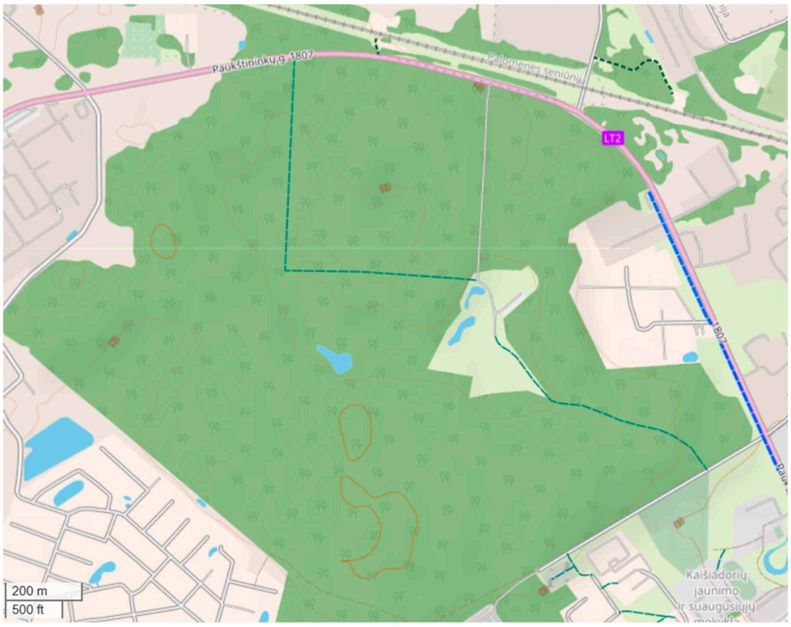

Figure 7. Kaišiadorys Forest Park Screenshot from OpenStreetMap [24]; map data © OpenStreetMap contributors.

Sampling site 9 - Central Park (Birštonas)

Birštonas is a balneological and spa resort town surrounded by extensive pine forests and the Nemunas River. Central Park, established within a 25-hectare forested area, serves as a recreational space frequented by numerous visitors (Figure 8). The park consists of managed lawns, mature predominantly broadleaved trees, and paved walking paths, with adjacent pinewoods and moist riverine zones. Ticks were collected from vegetation along paved paths within the park, as well as in the shrub–meadow ecotone near the river.

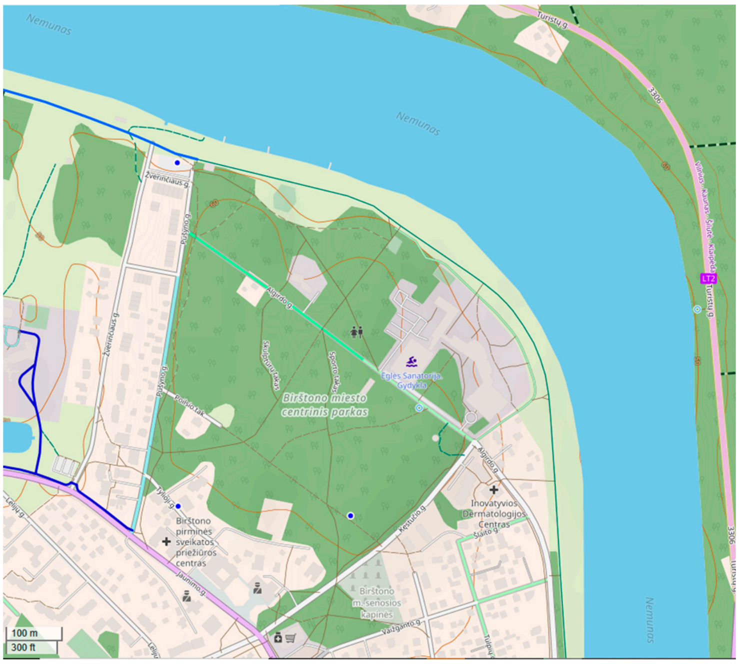

Figure 8. Central Park (Birštonas). Screenshot from OpenStreetMap [24]; map data © OpenStreetMap contributors.

Sampling site 10 - Nemunas Loops Regional Park (Paduoblis, Prienai district)

The Nemunas Loops Regional Park, covering 25,171 hectares, was established to protect the unique landscape of the large loops of the Nemunas River and the Punia pine forest, its natural ecosystem. The area combines remote natural zones with well-visited scenic sites (Figure 9). Ticks were collected near Paduoblis village, part of the regional park, where natural forested areas blend with private gardens and semi-cultivated spaces.

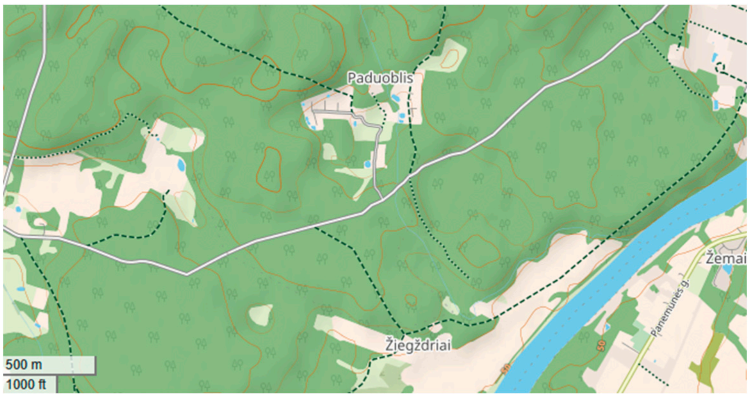

Figure 9. Nemuas Loops Regional Park (Prienai district). Screenshot from OpenStreetMap [24]; map data © OpenStreetMap contributors.

Sampling site 11 - Kaunas Street Cemetery (Kėdainiai)

The study site is located on the forested edge surrounding one of the Kėdainiai city's cemeteries, where a narrow pine stand borders urban infrastructure, pedestrian paths, and the cemetery grounds (Figure 10). This transitional zone between maintained cemetery vegetation and adjacent semi-natural pine woodland, with patches of unmanaged grassand and scattered shrubs, provides microhabitats suitable for questing ticks. Ticks were collected along the tree line, targeting both the open grassy margins and the interior of the pine stand.

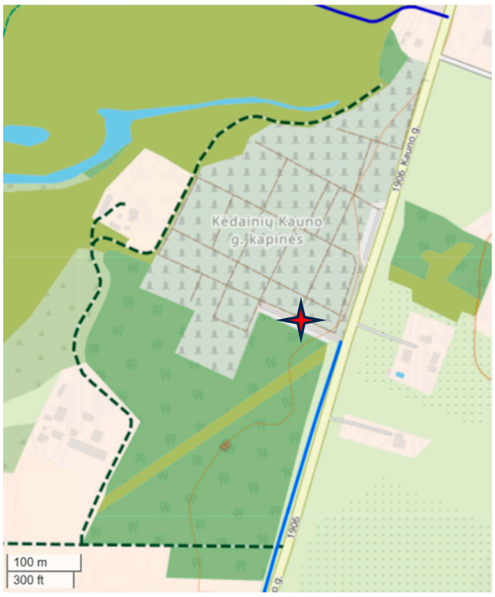

Figure 10. Kaunas Street Cemetery (Kėdainiai). Fig. 8. Central Park (Birštonas). Screenshot from OpenStreetMap [24]; map data © OpenStreetMap contributors.
