# Supplementary material for: Habitat-Specific Patterns of Tick-Borne Pathogens in Urban and Suburban Landscapes
Source: Pathogens. 2026 Apr 1;15(4):376. doi: 10.3390/pathogens15040376 (PMC13119425; doi:10.3390/pathogens15040376)
Supplement: Supplementary file 1 [file pathogens-15-00376-s001.zip › pathogens-4209368-Table S1.pdf]

Table S1. Location-specific prevalence of tick-borne pathogens in adults and nymphs of *Ixodes ricinus*

Location (No)		N	*Borrelia* spp.	*A.phagocytophilum*	*N. mikurensis*	*Rickettsia* spp.	*Babesia* spp.
			n (%)	n (%)	n (%)	n (%)	n (%)
Botanical garden (1)	F	93	23 (24.73)	5 (5.38)	7 (7.53)	12 (12.9)	0
	M	105	33 (31.43)	7 (6.67)	11 (10.48)	11 (10.48)	2 (1.90)
	N	103	30 (29.13)	1 (0.97)	13 (12.62)	11 (10.68)	1 (0.97)
Panemunė Pinewood park (2)	F	62	14 (22.58)	4 (6.45)	3 (4.84)	2 (3.23)	2 (3.23)
	M	41	4 (9.76)	2 (4.88)	1 (2.44)	2 (4.88)	2 (4.88)
	N	69	5 (7.25)	0	2 (2.90)	6 (8.70)	2 (2.90)
Kleboniškis forest park (3)	F	89	30 (33.71)	1 (1.12)	9 (10.11)	3 (3.37)	2 (2.25)
	M	75	19 (25.33)	10 (13.33)	12 (16.00)	6 (8.00)	2 (2.67)
	N	243	69 (28.40)	10 (4.12)	21 (8.64)	23 (9.47)	10 (4.12)
Zoological garden (5)	F	24	5 (20.83)	0	2 (8.33)	4 (16.67)	0
	M	22	5 (22.73)	1 (4.55)	3 (13.64)	3 (13.64)	0
	N	14	3 (21.43)	0	1 (7.14)	2 (14.29)	0
Academy campus (6)	F	19	4 (21.05)	1 (5.26)	3 (15.79)	3 (15.79)	0
	M	26	5 (19.23)	0	2 (7.69)	4 (15.38)	0
	N	11	1 (9.09)	0	1 (9.09)	1 (9.09)	0
III Fort of Kaunas Fortress (7)	F	2	1 (50.00)	0	0	0	0
	M	11	2 (18.18)	0	0	1 (9.09)	0
	N	13	3 (23.08)	0	0	2 (15.38)	0
Kaišiadorys Forest Park (8)	F	19	6 (31.58)	0	0	0	0
	M	24	9 (37.5)	1 (4.17)	0	0	0
	N	76	11 (14.47)	0	5 (6.58)	6 (7.89)	4 (5.26)
Central park (9)	F	97	29 (29.9)	6 (6.19)	7 (7.22)	14 (14.43)	2 (2.06)
	M	86	23 (26.74)	3 (3.49)	4 (4.65)	5 (5.81)	2 (2.33)
	N	13	0	0	0	0	0
Nemunas Loops Regional Park (10)	F	41	8 (19.51)	1 (2.44)	4 (9.76)	6 (14.63)	2 (4.88)
	M	37	7 (18.92)	2 (5.41)	4 (10.81)	3 (8.11)	1 (2.70)
	N	68	6 (8.82)	4 (5.88)	2 (2.94)	1 (1.47)	2 (2.94)

	F	28	13 (46.43)	1 (3.57)	6 (21.43)	0	2 (7.14)
Cemetery (11)	M	24	8 (33.33)	1 (4.17)	3 (12.5)	1 (4.17)	1 (4.17)
	N	2	0	0	0	1 (50.00)	0

No - number assigned to the sampling site; N – number of collected ticks; n – number of infected ticks, % – prevalence; F – females; M – males; N – nymphs
